# Influence of Mixing Order on the Synthesis of Geopolymer Concrete

**DOI:** 10.3390/polym14214777

**Published:** 2022-11-07

**Authors:** Timur Mukhametkaliyev, Md. Hazrat Ali, Viktor Kutugin, Olesya Savinova, Vladimir Vereschagin

**Affiliations:** 1 Department of Mechanical and Aerospace Engineering, School of Engineering and Digital Sciences, Nazarbayev University, Kabanbay Batyr Ave. 53, Astana 010000, Kazakhstan; 2 Research Laboratory “Refractory Non-Metallic and Silicate Materials”, National Research Tomsk Polytechnic University, 634050 Tomsk, Russia

**Keywords:** geopolymer, inorganic polymer, clay, concrete, fly ash, slag

## Abstract

Geopolymers are high-performance, cost-effective materials made from industrial waste that ideally fit the needs of 3D printing technology used in construction. The novelty of the present work lies in the investigation of methods to mix geopolymer concrete from fly ash (FA) class F, ground granulated blast furnace slag (GGBS), and raw calcined kaolin clay (RCKC) to determine the mixing procedure which provides the best mechanical strength and structural integrity. The experimental results show that aluminosilicates with different reaction parameters when mixed one after another provide the optimal results while the geopolymer concrete possesses the highest compressive strength and the denser structure. The results demonstrated that the reactivity of GGBS, FA, and RCKC increased for different depolymerization speeds of the selected aluminosilicates. This research will provide results on how to improve the mixing order for geopolymer synthesis for 3D printing demands. The highest compressive strength and denser structure of geopolymer concrete is achieved when each type of aluminosilicate is mixed with an alkaline medium separately.

## 1. Introduction

Three-dimensional construction printing (3DCP) offers revolutionary prospects of “smart” technologies for the construction industry with advantages that include formwork and mold-free manufacturing, increased geometrical freedom, improved safety in construction, reduction in construction waste, time, labor, and lower cost [1].

However, the introduction of 3DCP as a novel construction technology poses several challenges regarding material properties. Firstly, the printable material should be flowable enough to be pumped through the transporting system to the printing nozzle [2,3]. Secondly, the printable material should secure quick structural build-up to retain the designed shape and withstand its weight as well as deposited layers on the top right after the extrusion [2,3,4]. For ordinary Portland cement (OPC) concrete to be printable, a great number of additives, plasticizers, and stabilizing agents are required, which increase its initial cost. Moreover, the production of OPC triggers stronger CO_2_ emissions (8% of global CO_2_ emissions) [5]. The global standards of the modern construction industry imply the commitment to abate greenhouse gas emissions and decrease the energy-consuming process produced by OPC [5]. Therefore, current 3DCP technology needs to identify high-performing printable cementitious materials considering the need for controlled rheology, rapid hardening properties, and sustainable solutions.

Geopolymers have been introduced as a promising alternative to OPC with a staggering 90% lower CO_2_ footprint [6]. The distinctive advantage of a geopolymer over an OPC is that geopolymer cement can be synthesized at room temperature while OPC cement requires a four times higher amount of embodied energy for production [7].

Geopolymers are produced during the reaction of aluminosilicates with an alkaline solution of sodium or potassium silicate that creates a three-dimensional polymeric network. Geopolymers can be made from waste materials (FA, GGBS, etc.) which in turn reduces their carbon footprint [8]. The geopolymers possess exceptional performance in comparison with conventional cementitious materials by providing improved strength, durability, thermal resistance, and water resistance [9,10,11]. During mixing, the alkaline solution dissolves the aluminosilicates to release silicate and alumina monomers for polycondensation reactions afterward [7,12]. As a result, a rigid inorganic polymer network is produced with a potentially lower CO_2_ footprint, high early strength, and high thermal resistance [7,12]. Geopolymers are innovative solutions for industrial waste disposal in Kazakhstan’s massive mining and metallurgical industries. It was reported that more than 15 billion tons of industrial waste have already been accumulated in landfills, and this amount is annually replenished at dumps by another 1 billion [13]. The growing building industry of Kazakhstan demonstrates a significant rise in the fabrication of materials for construction purposes [14]. However, industrial waste is currently barely employed in the fabrication of construction materials [15,16,17]. The prices for construction materials in Kazakhstan rose by 25% in 2021 [18]. Therefore, there is an urgent need to use cost-effective material production based on recycling and circular economy business models. 

**Figure 1:**
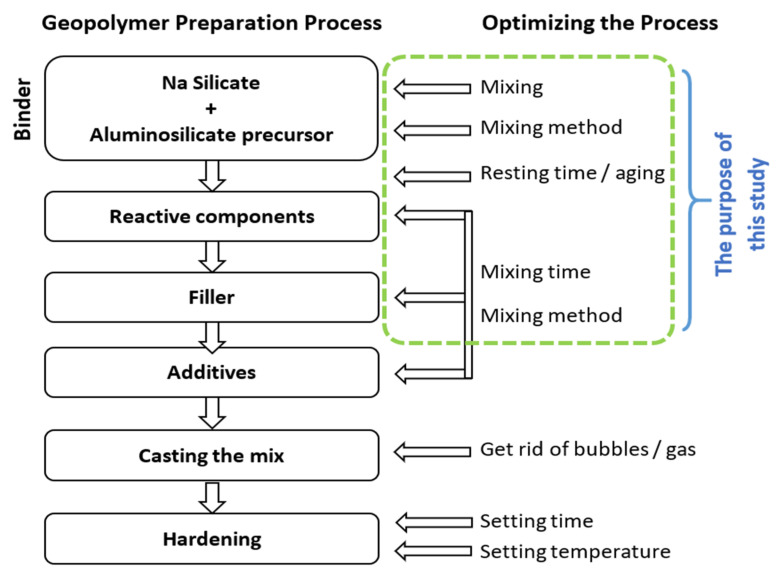
The geopolymer preparation process. Adapted from [5].

The scientific literature does not suggest any unified process for the manufacturing of geopolymer products. Machine learning approaches and modeling are proposed for predicting the compressive strength of FA and metakaolin-based geopolymer concrete [19,20,21]. The literature review shows that most of the research is concentrated on water-to-geopolymer solids ratios, alkaline-to-aluminosilicates ratios, and aggregates-to-geopolymer cement ratios but there is very scarce knowledge on the mixing time and mixing order of geopolymer precursors [12,22,23,24]. Mahmood described that a long time of mixing provides with denser structure and superior mechanical strength of geopolymer concrete [25]. Another author described that longer mixing time resulted in a lower slump of fresh concrete, and higher density and higher compressive strength of concrete [26]. It can be explained that in the presence of alkaline solutions, the continuous dissolution of silicate and aluminate molecules from FA, slag, and kaolin precursors induces polycondensation and the formation of geopolymer chains [27,28]. A longer mixing duration provides greater numbers of aluminate and silicate monomers to form geopolymer gels and geopolymer chains [29,30]. In most of the research papers, the dissolution and condensation are often simultaneous [31,32,33] because of the limited dissolution of species, and the various kinetics speed of different types of aluminosilicates that can lead to premature condensation. The inventor of geopolymers, Davidovits, reported that the manufacturing process must follow the geopolymerization reaction kinetics to obtain optimum efficiency [7]. Figure 1 displays the order in which products should be added to the mix. The alkali silicates must be given enough time to depolymerize the aluminosilicate precursor. Respecting this order allows the alkali silicate to have sufficient time to react with each component. If a more reactive ingredient is blended first, it risks absorbing more alkali silicates than it needs, which could create a deficit of alkali silicates for the remaining components and provide a slower or incomplete reaction [7]. To the knowledge of the authors based on existing the literature reviews, there was no research on how the order of adding the aluminosilicate precursors to the mix can influence the final mechanical properties of geopolymer concrete. Therefore, the time and order of mixing can have a direct consequence on the final polymerization mechanism.This observation constitutes the necessity of the current research paper. 

The three aluminosilicates used for geopolymer concrete fabrication in the present study are FA class F, RCKC, and GGBS. These aluminosilicates differ in elemental composition and in structure, which contributes to differences in reaction speed, polycondensation, as well the reactivity between them during geopolymerization. The previous studies suggest that the calcined kaolin clay is more reactive than the FA class F. Furthermore, the geopolymers fabricated from the FA class F showed a considerably lower compressive strength than samples made from calcined kaolin. This observation indicated that the production of a strong and durable geopolymer requires precursors of different reactivities to be mixed taking into consideration the time and speed of their reaction with an alkaline solution (Figure 1).

Kaolin or metakaolin precursors can provide comparatively higher Al content for geopolymer fabrication. Besides the use of industrial waste in geopolymer fabrication, it is a typical practice to apply calcined kaolin or metakaolin together with another aluminosilicate material to fabricate geopolymers [34,35,36,37]. It was observed that calcined kaolin or metakaolin could successfully provide a higher rate of geopolymerization therefore they were often used in combination with other precursor materials.

The primary objective of the research is to evaluate the effect of mixing multiple aluminosilicates one at a time and simultaneous mixing on:

1. Chemical composition;
2. Structure;
3. Morphology;
4. Mechanical strength.

## 2. Materials and Methods

**Figure 2:**
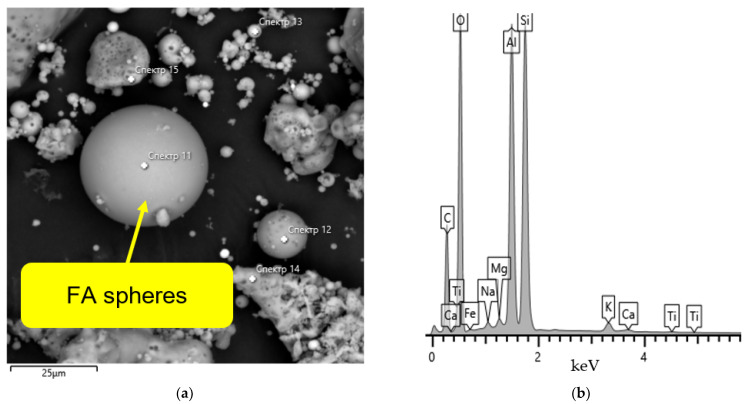
(**a**) SEM images and (**b**) EDX measurements of FA.

The FA was obtained from the thermal power plant of Karaganda city (Kazakhstan). The fly ash used in this study is of low calcium content and belongs to Class F fly ash as per ASTM C 618 [38]. Figure 2 shows the SEM images of FA and its chemical composition ratios from EDX measurements. Scanning electron microscopy (SEM) was employed to qualitatively analyze the differences in geopolymer samples’ microstructures and morphology. The sample surface was carbon-coated using an EMITECH K450X unit before SEM analysis to decrease noise and obtain images of high resolution. The SEM was conducted on Tescan Mira 3 LMU fitted with Ultim Max 40 energy dispersive detector and used an operating voltage of 20 kV, an aperture size of 1500 μm, with a regulated current of 4.6 nA. EDX analysis was made at 500× magnification within a 400 μm × 400 μm area. It can be seen that samples of FA contain silica and alumina and have similar chemical compositions. 

GGBS was collected from the ArcelorMittal company in Temirtau city (Kazakhstan). Raw kaolin clay (RKC) was obtained from the Kokshetau region. It was observed that kaolin clay can be activated by the calcination process because of transference of its crystalline structures into amorphous structures and increases in their chemical activities. Therefore, raw kaolin clay was calcined in a laboratory kiln at 750 °C. The alkaline activators are presented as a sodium hydroxide (99% NaOH) added to commercially available sodium silicate (molar ratio Si/Na = 3.32). The standard sand was used as the filler to produce the geopolymer samples.

The chemical composition of aluminosilicate components has several differences (Table 1). X-ray fluorescence analysis of major elements was carried out on a HORIBA XGT 7200 XRF microscope (Japan) under vacuum conditions and operating conditions of 0.5 mA for tube current and a voltage of 50 kV with a 1.2 mm spot size, and the acquisition time was 100 s. The RCKC contains more Al_2_O_3_ and GGBS. It was reported that calcined kaolin clay can provide five times more Al_2_O_3_ for dissolution in alkaline media than FA despite the fact that they can have similar amounts of Al_2_O_3_. The GGBS has substantially higher CaO contents than the RCKC and FA. Al_2_O_3_ and CaO are important elements because AlO silicate oligomers form rigid geopolymer networks [36]. The presence of calcium oxide in the source material strengthens the geopolymer network by Ca–Al-Si gel and results in room temperature settings [7,39].

The casting of the specimens was performed as follows:

1. Mix 1. FA was mixed with an alkaline solution for 10 min, and then followed by the addition of raw calcined kaolin clay (RCKC) that was mixed for 5 min, then GGBS was added, which had to be mixed for 3 min, and the last step was to add sand and mix for 3 min.
2. Mix 2. All aluminosilicates (FA, RCKC, GGBS) were mixed with the alkaline activator in one step for 18 min and after that, the standard sand was introduced to the mixture which then was mixed for 3 min.
3. Mix 3. All aluminosilicates (FA, RCKC, GGBS) and sand were mixed simultaneously with an alkaline activator for 21 min.

The developed concrete was cured in molds with dimensions of 30 × 30 × 30 mm. After that, it was covered with film to avoid evaporation. The curing of the samples was carried out at an ambient temperature to complete the geopolymerization. It should be noted that all weight ratios of aluminosilicates have been kept the same in all the mixing approaches.

Compressive strength and flexural strength tests were made to estimate the strength of all the mixtures according to ASTM [40,41]. Samples were tested on the 3rd day to observe the earliest structure formation and on the 28th day age of the concrete. Five samples in each mixture were tested by employing a PGM-100MG4 compression machine. The structures and phase compositions of the specimens were analyzed using X-ray diffraction. The diffractometer was set to the Bragg–Brentano configuration that implies radiation of Cu-Kα_1_ at 30 mA and 40 kV. PDF-4+ software was used to identify the phase composition.

Fourier transforms infrared (FTIR) spectra were obtained using a Shimadzu IR Prestige-21 infrared spectrometer. The IR spectra were registered in the middle-IR (MIR, 4000–400 cm^−1^) spectral region at a standard room temperature with a resolution of 4 cm^−1^. Each powered sample was mixed with potassium bromide powder (KBr, spectroscopic grade) in 1:60 proportion and ground down to form a uniform consistency in an agate pestle and mortar. Aliquots of the mixture were used to make pressed pellets for each sample. The results and following discussion are below.

## 3. Results and Discussion

### 3.1. Compressive Strength

**Figure 3:**
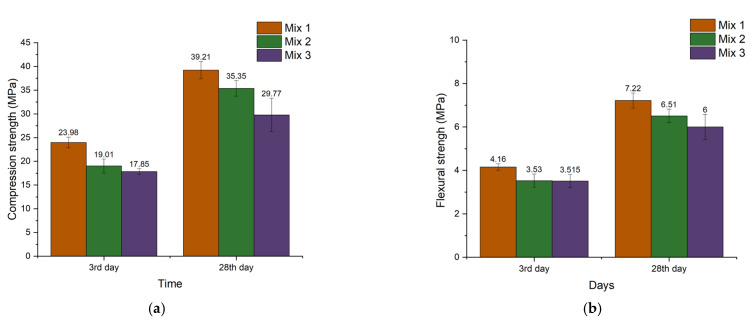
Compression strength and flexural strength. (**a**) Compressive strength; (**b**) flexural strength.

The compressive and flexural strength of each mix after 3 days of curing is shown in Figure 3. The Mix 1 sample demonstrates the highest compressive and flexural strength in comparison with other mixes. Mix 1 mix has higher mechanical strength than Mix 2 and Mix 3 which can be mainly attributed to a better dissolution of activated aluminosilicates that results in the increased amount of interconnected polymerized units as well as a less porous structure. A more detailed discussion on the resulting compressive strength for different mixing orders of aluminosilicates is discussed in the Microscopy section below.

The high compressive strength suggests that developed geopolymer concrete has high potential to be applicable in the use of structural 3D printing building materials, because of the early strength development [2].

### 3.2. Microscopy

**Figure 4:**
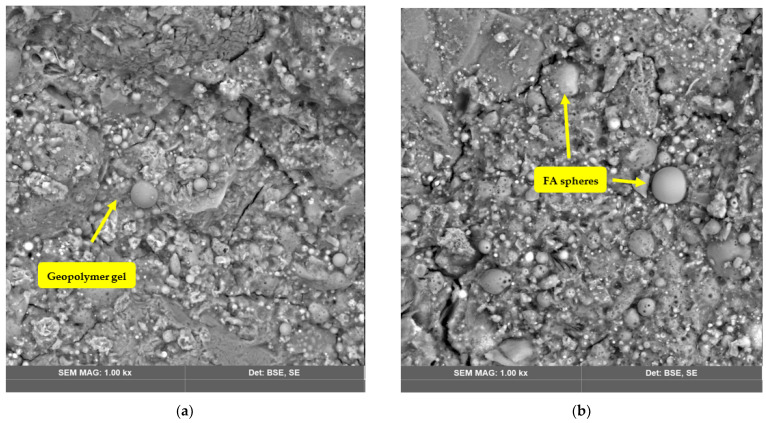

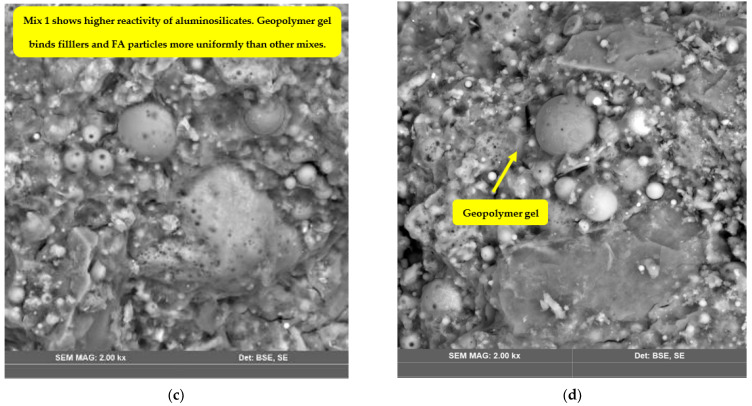
SEM images of Mix 1. (**a**,**c**) On images on the left were taken after 3 days of curing; (**b**,**d**) the images on the right were taken after 28 days of curing.

**Figure 5:**
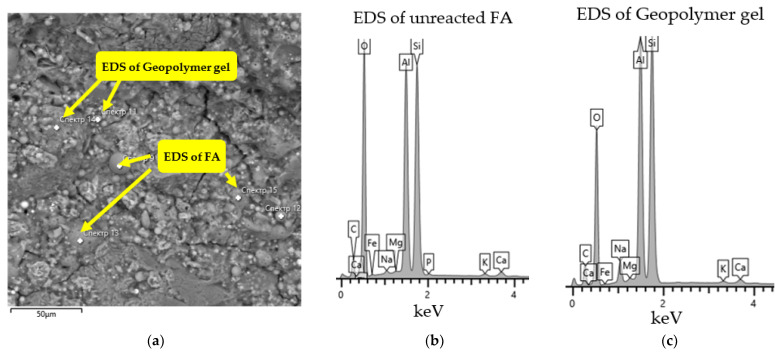
SEM image (**a**); EDS data of developed geopolymer concrete (Mix 1) where (**b**) is the EDS of unreacted FA, and (**c**) is the EDS of geopolymer gel.

**Figure 6:**
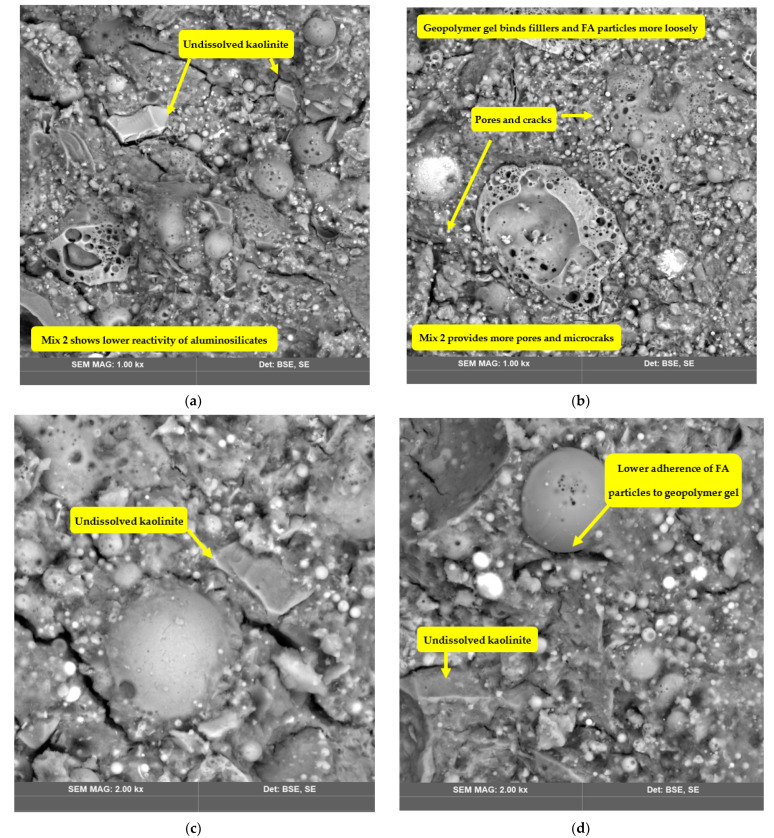
SEM images of Mix 2. The images on the left (**a**,**c**) were taken after 3 days of curing, and the images on the right (**b**,**d**) were taken after 28 days of curing.

Figure 1, Figure 4, Figure 5 and Figure 6 show the SEM images of the geopolymers fabricated from FA, RCKC, and GGBS. In Mix 1 (Figure 4a,b) the RCKC shows transformation to the gel phase, and the FA particles were left partly unreacted. 

EDS data in Figure 5 show the elemental composition of geopolymer gel (c) and unreacted FA particles (b). 

**Figure 7:**
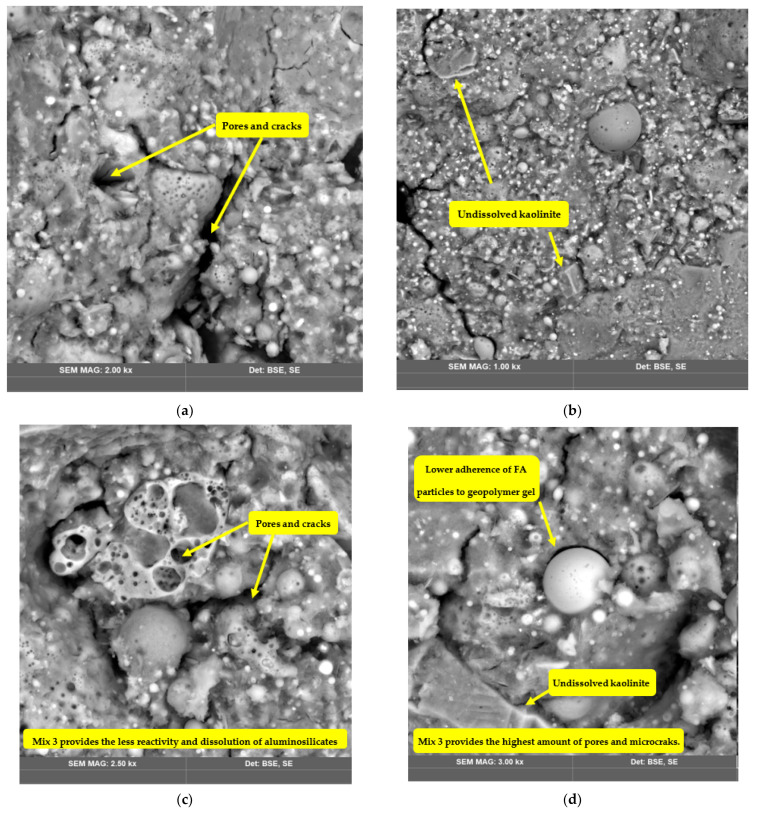
SEM images of Mix 3. The images on the left (**a**,**c**) were taken after 3 days of curing; the images on the right (**b**,**d**) were taken after 28 days of curing.

The geopolymer gel embeds Na in the polymer network which is confirmed by EDS data. It should be noted that the gel phase and source materials interact with each other. The RCKC’s higher reactivity enables ample interaction to occur to sufficiently increase the degree of geopolymerization. It was reported [42] in the pure metakaolin system after the geopolymerization process was completed that the lamellar structure of kaolinite undergoes disintegration and is transformed into dense gel formation. However, the simultaneous mixing of aluminosilicates particles in Mix 2 results in an incomplete reaction and dissolution of kaolinite. In Figure 6 and Figure 7 (Mix 2 and 3) there is a clear indication of undissolved kaolinite structures and lower adherence of FA particles to geopolymer gel in comparison with Mix 1. 

The lamellar structure of kaolinite could be detected in Figure 6 and Figure 7 which suggests incomplete dissolution of kaolinite. Residual or incompletely dissolved FA particles were presented in all the sets of photographs. However, images of Mix 1 showed a higher degree of dissolution of precursor materials than the other two groups and Mix 1 has a much denser matrix. A direct reflection of this in terms of mechanical properties is that the highest compressive strength is for the Mix 1 geopolymer.

### 3.3. X-ray Diffraction Analysis

**Figure 8:**
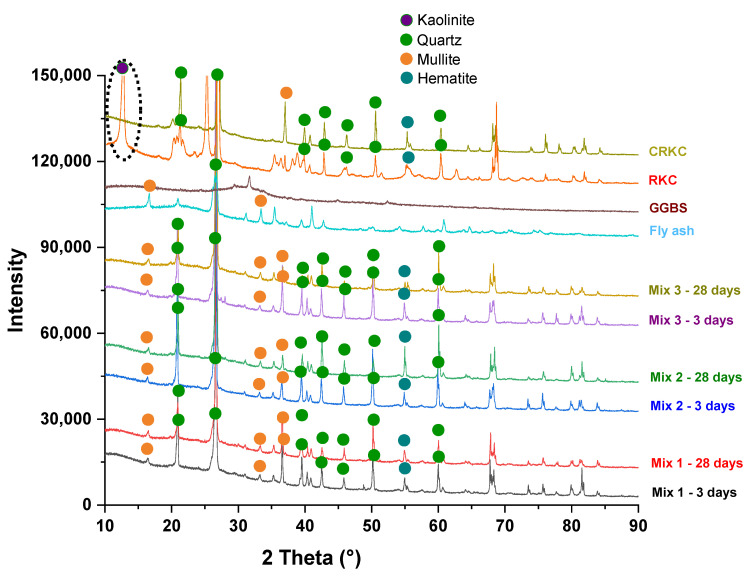
Integrated XRD patterns of developed geopolymer concrete samples.

Figure 8 depicts the X-ray diffraction patterns of RCKC, FA, GGBS, and geopolymer concrete samples with the ICDD PDF-2 database [43]. In contrast to the other samples, GGBS demonstrates a prevailing amorphous structure. The FA shows major peaks of mullite and quartz. It is stated that the calcination of GGBS, FA, and raw kaolin clay results in an increase in reactivity and geopolymerization [36,44,45]. Calcined materials mostly present an amorphous structure. The raw kaolin clay sample has a larger number of crystalline phases than the calcined sample. At 12° 2 Theta of RKC, a kaolinite structure was revealed which disappeared after calcination, confirming the transition of kaolinite to an amorphous state.

The characteristic background halo can be noticed for all geopolymer mixes from 15° to 33° 2 Theta. It is reported that this halo can indicate the amorphousness of the sample, which means that the proportion of dissolved fly ash in geopolymer increases [46]. By comparing the XRD patterns of both geopolymer and raw materials, it was observed that a broader hump in the range of 27–29° (2 Theta) in XRD patterns of geopolymers can primarily be the result of the extensive presence of nano-crystal size zeolites [47] as well as part of the partially reacted crystalline quartz phase from filler at around 27° (2 Theta) (Figure 8).

### 3.4. FTIR Spectroscopy

**Figure 9:**
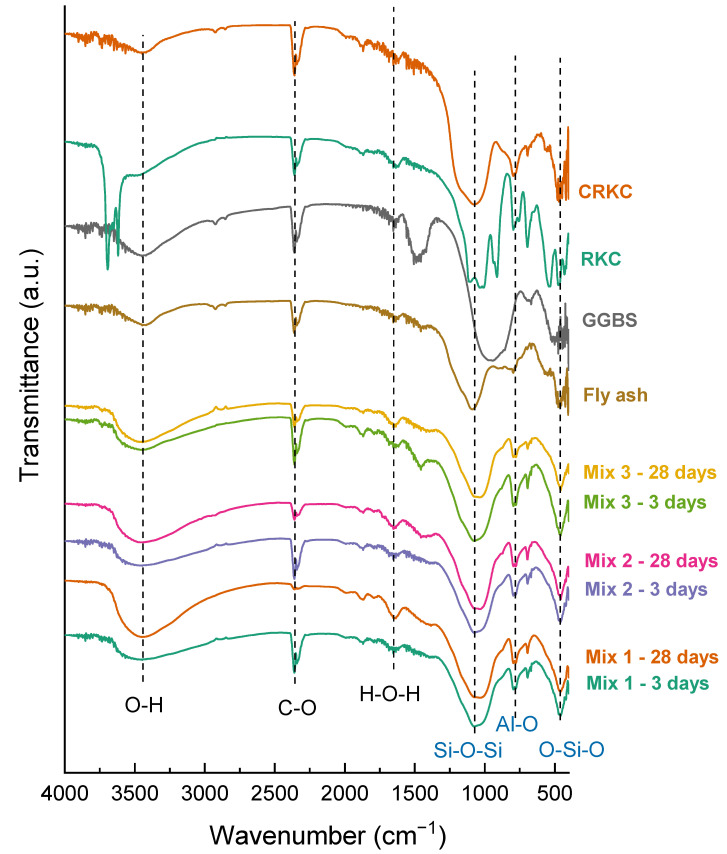
FTIR spectrums of developed geopolymer concrete samples.

The obtained FTIR graphs of the source materials and geopolymer concrete samples that were made under different mixing conditions are presented in Figure 9. The primary elements involved in the framework structure of geopolymers are Si, Al, O, H, and alkali cations whose chemical bonds can be successfully measured by FTIR. The presence and appearance of bonds (in terms of bands, shoulder, or sharp peaks) provide useful information on the formation and microstructure of geopolymers.

The most relevant bands are observed in the region of 1040–1075 cm^−1^ and represent the asymmetrical stretching band of Si-O-T (T, Si, or Al). In geopolymer sets cured for 28 days, the Si-O-T group was discovered to change from 1075 cm^−1^ to 1040 cm^−1^, indicating the development of amorphous silicon aluminate gels which is consistent with the results of mechanical property measurements.

The band at 420–470 cm^−1^ is attributed to the vibrational modes of the bending vibration of the Si-O group that appears weaker in samples that were cured for 28 days. The band at 610–615 cm^−1^ appearing on Mix 1 and Mix 2 spectra is correlated with the vibrational mode of AlO_4_ in the 4-coordinated position [7]. 

The peak at 694 cm^−1^ is the symmetrical stretching vibration of Si-O groups that depicts the quartz in the precursor materials [48]. The peaks at 777–797 cm^−1^ represent the symmetrical stretching vibrations of the Si-O-Si. 

The band at 870–885 cm^−1^ and the peaks of the 1400–1450 cm^−1^ range belong to the CO_3_^2-^ a group of the bending vibration and the asymmetrical stretching vibration. The peak intensity is the highest in Mix 3 in both samples cured for 3 and 28 days suggesting that mixing procedure №3 (c) causes the carbonization of the geopolymer and results in a decrease in strength. The peaks related to gaseous CO_2_ are also visible at about 2341 cm^−1^. Carbonization occurs only in Mix 3, probably due to the formation of sodium carbonate which causes the deterioration in strength properties, which can be explained by the fact that carbonates have perfect cleavage (easily split along crystallographic planes), and this can perhaps affect the mechanical strength. 

Bands in the regions of 3450 cm^−1^ demonstrate the stretching vibrations of the external and internal O-H groups that correspond to structural and interlayer water. The band at 1650 cm^−1^ belongs to the H-O-H bending vibration attributed to the presence of H_2_O molecules. The reformations of H-O-H and O-H vibration bands in the sample sets cured for 3 and 28 days confirm the development of aluminosilicate networks in the geopolymers [49].

**Figure 10:**
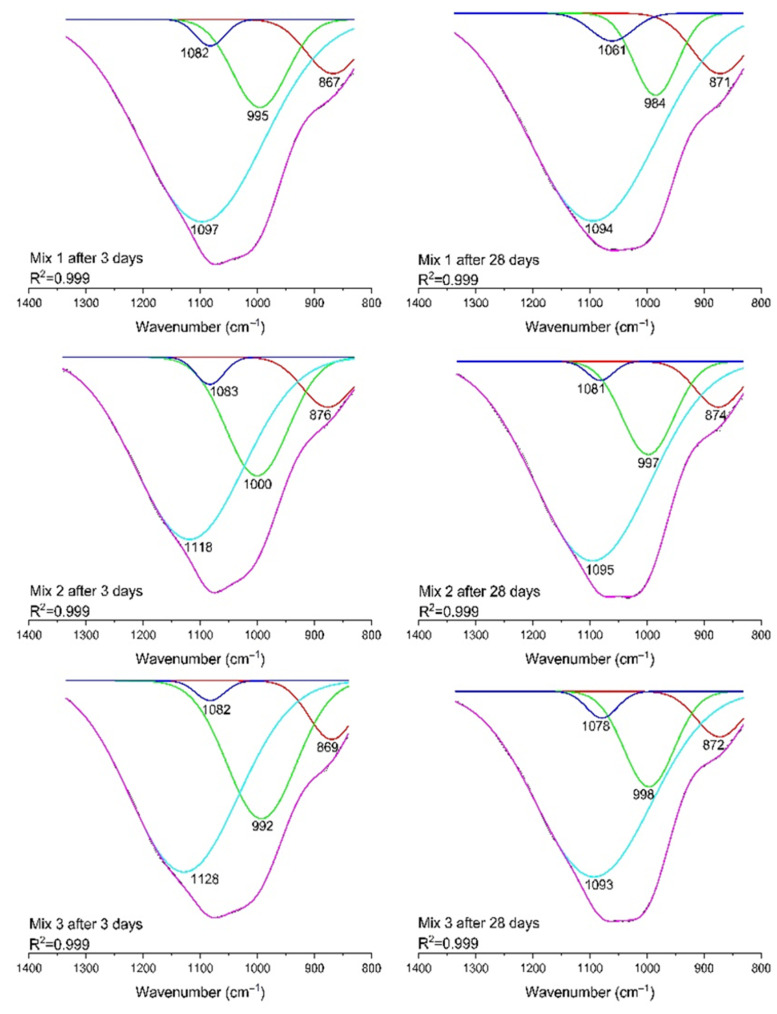
FTIR spectrum deconvolution of geopolymers.

The most relevant bands are observed in the region of 800–1340 cm^−1^ and represent the asymmetrical stretching band of Si-O-T (T = Si or Al). It is considered that the increase in Si-O-Al bond concentration entails a reduction in wavenumber [50]. In geopolymer sets cured for 28 days, the Si-O-T group was discovered to change from 1075 cm^−1^ to 1040 cm^−1^, indicating the development of amorphous silicon aluminate gels which is consistent with the results of mechanical property measurements. For a detailed interpretation of the aluminosilicate gel structural changes, the FTIR spectra were deconvoluted within the range of 830–1340 cm^−1^ (Figure 10). A peak at 867–876 cm^−1^ is considered to be the vibration of Si-OH at the end of the aluminosilicate framework [51]. A peak in the range of 984–1000 cm^−1^ could be attributed to Si-O-T in the three-dimensional framework of the aluminosilicate gel [51,52]. Peaks at 1061–1083 cm^−1^ and 1093–1128 cm^−1^ are assigned to the symmetric and asymmetric vibrations of unreacted silica present in the matrix [51].

## 4. Conclusions

In this study, geopolymer concrete samples from FA, RCKC, and GGBS are experimentally investigated, with different mixing procedure methods of the aluminosilicates and their effects on chemical composition, structure, morphology, and mechanical strength being studied. The developed samples were examined with SEM, EDX, XRD, and FTIR. Both the flexural and compressive strengths of developed geopolymer concrete were evaluated. The analysis of the experimental results has led to the following conclusion:

(1) The mixing procedure of aluminosilicate precursors, which allows one ingredient to mix at a time, yields a higher degree of geopolymerization which results in a denser structure, and higher mechanical strength. The compressive strength of geopolymer concrete is increased by 31.7% and the flexural strength is increased by 20.3%. The compressive strength of geopolymers is inevitably correlated with the internal microstructure shown by SEM images, which in turn is formed by the polycondensation of multiple dissolution products of raw materials.
(2) The simultaneous mixing of FA, RCKC, GGBS, and filler inhibits the reaction rate and reduces the average reactivity of the raw materials. The solid particles of kaolinite and FA that did not react during the dissolution of aluminosilicates are not completely connected with the matrix, and the residual pores and gaps in the structure around them can result in a decrease in mechanical performance. A more continuous and denser geopolymer gel phase is found in Mix 1, while Mix 2 and Mix 3 appear to have a more bulky, irregular structure with a larger distribution of pores and cracks.
(3) The geopolymer mix design needs fully yield the potential of geopolymerization leading to the best performance and the highest mechanical strength. Therefore, the results of this study indicate that exploration of other mixing parameters such as mixing time, mixing speed (rotation per minute), and their influence on geopolymer performance are important for further research plans.
(4) The separate mixing of aluminosilicates may not be practical in large-scale applications due to time-consuming factors, but it can be stated that the addition of aggregates after geopolymer paste preparation (Mix 2) provides superior mechanical strength and structure in the simultaneous mixing of geopolymer precursors and filler (Mix 3).

The next study aims to investigate the influence of the mixing order of aluminosilicates on the extrusion properties of geopolymer concrete to be used in concrete 3D printing applications.

## Figures and Tables

**Table 1:** Chemical composition of aluminosilicates.

Chemical Composition (wt. %)	FA	GGBS	RCKC
SiO_2_	65.605	31.0106	63.0988
TiO_2_	1.2755	0.6936	0.5504
Al_2_O_3_	25.0715	8.9084	28.9502
MgO	0.5323	10.7629	0.2343
CaO	2.5266	26.6471	1.9797
Na_2_O	0.6127	0.6764	0.7943
K_2_O	0.485	0.8022	0.9348
P_2_O_5_	0.2941	0.1057	0.1057

## Data Availability

Not applicable.

## References

[1] Vantyghem G., De Corte W., Shakour E., Amir O. (2020). 3D printing of a post-tensioned concrete girder designed by topology optimization. Autom. Constr..

[2] Buswell R.A., Leal de Silva W.R., Jones S.Z., Dirrenberger J. (2018). 3D printing using concrete extrusion: A roadmap for research. Cem. Concr. Res..

[3] Khan M.A. (2020). Mix suitable for concrete 3D printing: A review. Mater. Today Proc..

[4] Kristombu Baduge S., Navaratnam S., Abu-Zidan Y., McCormack T., Nguyen K., Mendis P., Zhang G., Aye L. (2021). Improving performance of additive manufactured (3D printed) concrete: A review on material mix design, processing, interlayer bonding, and reinforcing methods. Structures.

[5] Worrell E., Price L., Martin N., Hendriks C., Meida L.O. (2001). Carbon dioxide emissions from the global cement industry. Annu. Rev. Energy Environ..

[6] Valente M., Sambucci M., Sibai A. (2021). Geopolymers vs. Cement Matrix Materials: How Nanofiller Can Help a Sustainability Approach for Smart Construction Applications—A Review. Nanomaterials.

[7] Davidovits J. (2008). Geopolymer Chemistry and Applications.

[8] Yang K.-H., Song J.-K., Song K.-I. (2013). Assessment of CO_2_ reduction of alkali-activated concrete. J. Clean. Prod..

[9] Bakharev T. (2005). Durability of geopolymer materials in sodium and magnesium sulfate solutions. Cem. Concr. Res..

[10] Sumajouw M.D.J., Rangan B.V. (2006). Low-Calcium Fly Ash-Based Geopolymer Concrete: Reinforced Beams and Columns.

[11] Kong D.L.Y., Sanjayan J.G. (2008). Damage behavior of geopolymer composites exposed to elevated temperatures. Cem. Concr. Compos..

[12] Ranjbar N., Kuenzel C., Spangenberg J., Mehrali M. (2020). Hardening evolution of geopolymers from setting to equilibrium: A review. Cem. Concr. Compos..

[13] Egov.kz Public Services and Online Information. https://egov.kz/cms/en/articles/ecology/waste_reduction_recycling_and_reuse.

[14] PrimeMinister.kz (2020). Production Volume for Building Materials Increased 3 Times. Prime Minister Press Service of the Republic of Kazakhstan.

[15] Zhou Y., Weng Y., Li L., Hu B., Huang X., Zhu Z. (2022). Recycled GFRP Aggregate Concrete Considering Aggregate Grading: Compressive Behavior and Stress-Strain Modeling. Polymers.

[16] Liang Z., Hu Z., Zhou Y., Wu Y., Zhou X., Hu B., Guo M. (2022). Improving recycled aggregate concrete by compression casting and nano-silica. Nanotechnol. Rev..

[17] Zhou Y., Gao H., Hu Z., Qiu Y., Guo M., Huang X., Hu B. (2021). Ductile, durable, and reliable alternative to FRP bars for reinforcing seawater sea-sand recycled concrete beams: Steel/FRP composite bars. Constr. Build. Mater..

[18] KazTAG (2021). Prices for Construction Materials Increased by 25% in Kazakhstan. https://kaztag.kz/en/news/prices-for-construction-materials-increased-by-25-in-kazakhstan.

[19] Nguyen K.T., Nguyen Q.D., Le T.A., Shin J., Lee K. (2020). Analyzing the compressive strength of green fly ash based geopolymer concrete using experiment and machine learning approaches. Constr. Build. Mater..

[20] Ahmed H.U., Mostafa R.R., Mohammed A., Sihag P., Qadir A. (2022). Support vector regression (SVR) and grey wolf optimization (GWO) to predict the compressive strength of GGBFS-based geopolymer concrete. Neural Comput. Appl..

[21] Raza M.H., Zhong R.Y. (2022). A sustainable roadmap for additive manufacturing using geopolymers in construction industry. Resour. Conserv. Recycl..

[22] Imtiaz L., Rehman S.K.U., Ali Memon S., Khizar Khan M., Faisal Javed M. (2020). A Review of Recent Developments and Advances in Eco-Friendly Geopolymer Concrete. Appl. Sci..

[23] Amran M., Debbarma S., Ozbakkaloglu T. (2021). Fly ash-based eco-friendly geopolymer concrete: A critical review of the long-term durability properties. Constr. Build. Mater..

[24] Cong P., Cheng Y. (2021). Advances in geopolymer materials: A comprehensive review. J. Traffic Transp. Eng..

[25] Mahmood A.H., Foster S.J., Castel A. (2021). Effects of mixing duration on engineering properties of geopolymer concrete. Constr. Build. Mater..

[26] Hardjito D., Sumajouw D.M.J. Introducing fly ash-based geopolymer concrete: Manufacture and engineering properties. Proceedings of the 30th Conference on Our World in Concrete & Structures.

[27] Hajimohammadi A., Provis J.L., van Deventer J.S.J. (2010). Effect of Alumina Release Rate on the Mechanism of Geopolymer Gel Formation. Chem. Mater..

[28] Fernandez-Jimenez A.M., Palomo A., Lopez-Hombrados C. (2006). Engineering Properties of Alkali-Activated Fly Ash Concrete. ACI Mater. J..

[29] Gao X., Yu Q.L., Brouwers H.J.H. (2015). Reaction kinetics, gel character and strength of ambient temperature cured alkali activated slag–fly ash blends. Constr. Build. Mater..

[30] Chithiraputhiran S., Neithalath N. (2013). Isothermal reaction kinetics and temperature dependence of alkali activation of slag, fly ash and their blends. Constr. Build. Mater..

[31] Duxson P., Fernández-Jiménez A., Provis J.L., Lukey G.C., Palomo A., van Deventer J.S.J. (2007). Geopolymer technology: The current state of the art. J. Mater. Sci..

[32] Wan Q., Rao F., Song S., García R.E., Estrella R.M., Patiño C.L., Zhang Y. (2017). Geopolymerization reaction, microstructure and simulation of metakaolin-based geopolymers at extended Si/Al ratios. Cem. Concr. Compos..

[33] Lee W.K.W., van Deventer J.S.J. (2002). Structural reorganisation of class F fly ash in alkaline silicate solutions. Colloids Surf. A Physicochem. Eng. Asp..

[34] Van Jaarsveld J.G.S., Lukey G.C., Van Deventer J.S.J. (2000). The stabilisation of mine tailings by reactive geopolymerisation. Publ. Australas. Inst. Min. Metall..

[35] Van Jaarsveld J.G.S., Van Deventer J.S.J., Schwartzman A. (1999). The potential use of geopolymeric materials to immobilise toxic metals: Part II. Material and leaching characteristics. Miner. Eng..

[36] Xu H., Van Deventer J.S.J. (2000). The geopolymerisation of alumino-silicate minerals. Int. J. Miner. Process..

[37] Xu H., van Deventer J.S.J., Lukey G.C. (2001). Effect of Alkali Metals on the Preferential Geopolymerization of Stilbite/Kaolinite Mixtures. Ind. Eng. Chem. Res..

[38] (2019). Standard Specification for Coal Fly Ash and Raw or Calcined Natural Pozzolan for Use in Concrete.

[39] Yip C.K., Lukey G.C., van Deventer Dean J.S.J. (2006). Effect of Blast Furnace Slag Addition on Microstructure and Properties of Metakaolinite Geopolymeric Materials. Advances in Ceramic Matrix Composites IX.

[40] (2016). Standard Test Method for Compressive Strength of Cylindrical Concrete Specimens.

[41] (2016). Standard Test Method for Flexural Strength of Concrete (Using Simple Beam With Center-Point Loading).

[42] Yuan L., Ma Y., Zhang J., Men J., Sun T., Zhao H., Wu H., Wang H., Dai S. (2022). Orthogonal analysis and mechanism of compressive strength and microstructure of the metakaolin-fly ash geopolymer. Case Stud. Constr. Mater..

[43] Gates-Rector S., Blanton T. (2019). The Powder Diffraction File: A Quality Materials Characterization Database. Powder Diffr..

[44] Barbosa V.F.F., MacKenzie K.J.D., Thaumaturgo C. (2000). Synthesis and characterisation of materials based on inorganic polymers of alumina and silica: Sodium polysialate polymers. Int. J. Inorg. Mater..

[45] Palomo A., Grutzeck M.W., Blanco M.T. (1999). Alkali-activated fly ashes: A cement for the future. Cem. Concr. Res..

[46] Nenadović S., Gulicovski J., Mirković M., Kljajević L., Bošković I., Vukčević M., Nenadović M. (2022). Structural, Mechanical and Chemical Properties of Low Content Carbon Geopolymer. Sustainability.

[47] Provis J.L., Lukey G.C., van Deventer J.S.J. (2005). Do Geopolymers Actually Contain Nanocrystalline Zeolites? A Reexamination of Existing Results. Chem. Mater..

[48] Sha D., Pan B., Sun Y. (2020). A novel raw material for geopolymers: Coal-based synthetic natural gas slag. J. Clean. Prod..

[49] Sha D., Pan B., Sun Y. (2020). Investigation on mechanical properties and microstructure of coal-based synthetic natural gas slag (CSNGS) geopolymer. Constr. Build. Mater..

[50] Zhang Z., Wang H., Provis J.L., Bullen F., Reid A., Zhu Y. (2012). Quantitative kinetic and structural analysis of geopolymers. Part 1. The activation of metakaolin with sodium hydroxide. Thermochim. Acta.

[51] Li J., Tao Y., Zhuang E., Cui X., Yu K., Yu B., Boluk Y., Bindiganavile V., Chen Z., Yi C. (2022). Optimal amorphous oxide ratios and multifactor models for binary geopolymers from metakaolin blended with substantial sugarcane bagasse ash. J. Clean. Prod..

[52] Silva A.M.B., Queiroz C.M., Agathopoulos S., Correia R.N., Fernandes M.H.V., Oliveira J.M. (2011). Structure of SiO_2_–MgO–Na_2_O glasses by FTIR, Raman and 29Si MAS NMR. J. Mol. Struct..

